# The Intrarenal Reflux Diagnosed by Contrast-Enhanced Voiding Urosonography (ceVUS): A Reason for the Reclassification of Vesicoureteral Reflux and New Therapeutic Approach?

**DOI:** 10.3390/biomedicines12051015

**Published:** 2024-05-05

**Authors:** Marijan Saraga, Mirna Saraga-Babić, Adela Arapović, Katarina Vukojević, Zenon Pogorelić, Ana Simičić Majce

**Affiliations:** 1 School of Medicine, University of Split, 21000 Split, Croatia; zpogorelic@kbsplit.hr (Z.P.); 2 Department of Anatomy, Histology and Embryology, School of Medicine, University of Split, 21000 Split, Croatia; msb@mefst.hr; 3 Department of Pediatric, University Hospital of Split, 21000 Split, Croatia; aarapov@kbsplit.hr (A.A.); asimmajce@kbsplit.hr (A.S.M.); 4 Department of Pediatric Surgery, University Hospital of Split, 21000 Split, Croatia

**Keywords:** vesico-ureteral reflux, intrarenal reflux, VUR nephropathy, contrast-enhanced voiding urosonography, DMSA scintigraphy, voiding cystourethrography, children

## Abstract

Vesicoureteral reflux (VUR) is defined as the urine backflow from the urinary bladder to the pyelo-caliceal system. In contrast, intrarenal reflux (IRR) is the backflow of urine from the renal calyces into the tubulointerstitial space. VURs, particularly those associated with IRR can result in reflux nephropathy when accompanied by urinary tract infection (UTI). The prevalence of IRR in patients with diagnosed VUR is 1–11% when using voiding cystourethrography (VCUG), while 11.9–61% when applying the contrast-enhanced voiding urosonography (ceVUS). The presence of IRR diagnosed by VCUG often correlates with parenchymal scars, when diagnosed by a 99mTc dimercaptosuccinic acid scan (DMSA scan), mostly in kidneys with high-grade VURs, and when diagnosed by ceVUS, it correlates with the wide spectrum of parenchymal changes on DMSA scan. The study performed by both ceVUS and DMSA scans showed IRRs associated with non-dilated VURs in 21% of all detected VURs. A significant difference regarding the existence of parenchymal damage was disclosed between the IRR-associated and IRR-non-associated VURs. A higher portion of parenchymal changes existed in the IRR-associated VURs, regardless of the VUR grade. That means that kidneys with IRR-associated VURs represent the high-risk group of VURs, which must be considered in the future classification of VURs. When using ceVUS, 62% of places where IRR was found were still unaffected by parenchymal changes. That was the basis for our recommendation of preventive use of long-term antibiotic prophylaxis until the IRR disappearance, regardless of the VUR grade. We propose a new classification of VURs using the ceVUS method, in which each VUR grade is subdivided based on the presence of an IRR.

## 1. Introduction

### 1.1. Definitions

Intrarenal reflux (IRR) is a well-known phenomenon that sometimes occurs in some patients with vesicoureteral reflux (VUR). The VUR is defined as the backflow of urine from the urinary bladder to the pyelo-caliceal system through the dysfunctional vesicoureteric junction. Conversely, the IRR is defined as the backflow of urine from the renal calyces into the tubulointerstitial space through the papillary ducts.

### 1.2. Prevalence of VUR

The prevalence of VUR is approximately 1–2% in healthy children, 30–50% in children with recurrent febrile urinary tract infection (UTI), 1167% in asymptomatic siblings of patients with VUR, and 65% in children whose patients have dilated VURs [1,2,3,4,5].

### 1.3. The Diagnostic Methods for the Detection of VUR and Its Classification

The VUR is usually diagnosed by voiding cystourethrography (VCUG), and by contrast-enhanced voiding urosonography (ce-VUS) according to the protocols. Both procedures are performed by catheterization of the urinary bladder and by filling the bladder with contrast media. The procedures are followed by observing doctors and technicians during the filling and voiding phases, and all phases are documented and recorded. The main difference between those two methods is that VCUG bears radiation for the patient, while ce-VUS is radiation-free [6,7,8,9,10,11,12,13,14]. The VUR can be classified into five grades by both MCUG and ceVUS techniques. The VURs of grades I and II are considered as non-dilated VURs, grade III as a mildly dilated VUR, and VURs of grades IV and V as highly dilated VURs, respectively. Both diagnostic techniques have shown a high level of concordance regarding the assessment of VUR existence and grades [6,15,16,17,18,19,20,21,22,23,24]. If we take into account the high sensitivity of the ce-VUS in the diagnosis of VUR and the absence of ionizing burden to patients, ce-VUS seems to be a very acceptable choice for the diagnosis of VUR in the future [21,25]. The diagnostic sensitivity and specificity of ceVUS are superior to VCUG. A recent meta-analysis has clearly shown that ceVUS has a sensitivity of 93% and a specificity of 91% [26].

### 1.4. Consequences of VUR

VUR may be the cause of reflux nephropathy in approximately 12.5% of all patients and 25% of children with end-stage renal disease (ESRD). The presence of VUR can be accompanied by renal parenchymal defects, diagnosed by intravenous urography (IVU) in the past or present by 99mTc dimercaptosuccinic acid scan (DMSA scan) according to the protocols [7,9,27,28,29,30,31,32,33,34,35,36,37].

### 1.5. The Role of DMSA Scan in the Assessment of UTI and Reflux Nephropathy

Some groups of authors proved that 37–40% of kidneys with VUR had scars on the DMSA scan, but 6% of kidneys without VUR also had scars after febrile UTI and vice versa. The study by Gleeson FV et al. showed that 62% of all children with scars had VUR, especially children younger than 1 year of age [38,39]. In the acute febrile UTI, 26–78% of kidneys had a pathologic finding on a DMSA scan, while 38–52% of those findings remained pathologically changed several months after UTI on the control DMSA scan [30,40]. However, the presence of VUR was irrelevant if pyelonephritis had been already present in the renal parenchyma [40]. Generally, that means that only 13% of kidneys affected by pyelonephritis will develop permanent parenchymal changes following an acute UTI [32]. In kidneys with permanent changes 48% had VUR, while in the group with normal DMSA scans, VUR was present only in 12% of cases [32]. The concomitant increase of cortical defects associated with the increased grade of VUR was shown in recent studies [7,41,42].

Even though it is not easy to determine what factor is more important for the development of parenchymal scars in kidneys with UTI, it was shown that kidneys with a high grade of VUR and UTI developed scars in 43% of kidneys, while in the group with UTI and low grade of VUR, it was only 8%. Generally, the incidence of parenchymal changes in kidneys with UTI and VUR was shown to be 37–68%, while that was only 6–33% in kidneys with only UTI but without VUR. That speaks in favor of the important role of VUR in the development of renal parenchymal defects [7,31,32,38,39,40,41,42,43]. Some authors suspected that VUR nephropathy could be the end consequence of „silent“ urinary tract infections accompanied by VUR in the postnatal period or a hereditary phenomenon that goes without a history of UTI [44,45,46]. On the other hand, the reflux nephropathy which increased over time, together with recurrences of UTI, was associated with the highest number of scars in the group of older children. This implies that there are several known contributing factors, such as UTI or slow inflammatory processes that can lead to focal parenchymal defects. These facts open numerous questions about whether the critical factor for the development of parenchymal scars is an infection, the slow inflammatory process, something else like the existence of IRR, or all those factors together might be involved. The DMSA findings sometimes are not easy to interpret due to the possible interobserver variabilities in interpretation. Therefore, before the establishment of firm diagnostic standards for the interpretation of DMSA scans, it would be good if the findings were interpreted by at least two independent nuclear medicine specialists [7,9,37,43].

### 1.6. The Management Strategy of VUR

The management of VUR is not unique all over the world. There are still controversies regarding the treatment of patients with VUR. Generally, the VUR can be treated by antibiotics if the child has an acute UTI. After the treatment of acute UTI, some children will be followed up on, and some will receive long-term antibiotic prophylaxis (LTAP) until the spontaneous resolution of VUR or until the surgical treatment. The indications for LTAP are as follows: young age (younger than 12 months), female sex, bilateral dilated VUR, bladder-bowel dysfunction (BBD), and uncircumcised males. The usually used antibiotics for LTAP are trimethoprim–sulfamethoxazole, amoxicillin, and nitrofurantoin at a quarter to half of the regular therapeutic dose. The optimal timing for LTAP is controversial and is individualized to each case [47]. One recent study recommended the personalized approach to LTAP recommended by some other previous studies [48]. In the cases of LTPA noncompliance, breakthrough febrile UTIs despite LTAP, or symptomatic VUR, surgical treatment could be applied. The standard of treatment is an endoscopic approach by the subureteric injection of a bulking agent with an overall success rate of 85% or by an opened surgical approach with an overall success rate of 92–98% [49]. The newest recommendations were published by the European Association of Urology/European Society of Paediatric Urology—Paediatric Guidelines on Vesicoureteral Reflux in Children [47].

## 2. Intrarenal Reflux

**Figure 1:**
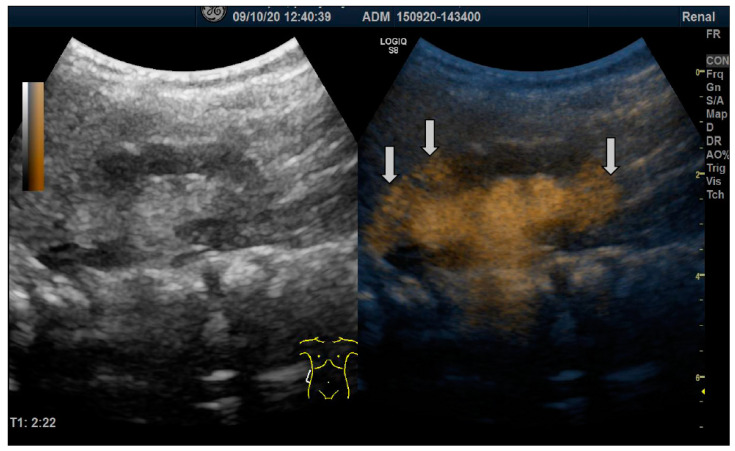
The duplex view of the right kidney. On the left side of the figure, the right kidney is visible in the B-mode. On the right side of the picture, the ultrasound contrast media is visible in the mildly dilated pyelo-caliceal system with convex calyces. In the upper and lower poles, the contrast media is visible in the renal parenchyma (arrows). The diagnosis is VUR grade III with intrarenal reflux in the upper and lower pole.

The first description of IRR was performed in 1965 by Brodeur et al. and was diagnosed by barium sulfate contrast media. After that, IRR was routinely diagnosed for a long time by VCUG [50]. The presence of IRR in patients with VUR was shown to be closely related to the existence of compound papillae type II and III in the kidney, as previously described in young pigs [51]. While the simple papillae are convex and have oblique and closed apertures, which are not permeable for the refluxing urine, the compound papillae type II and III are permeable for the backflowed urine, as they have a specific concave morphology with widely opened ductal apertures. These types of papillae are distributed mostly in the upper and lower kidney poles, while rarely in the mid-zone of the kidney. Those permeable papillae are presented as IRRs during the VCUG or ceVUS, as the sites of IRRs are located in the compound papillae type II or III of refluxing kidneys (Figure 1) [1,33,51]. The possible reason for the occurrence of IRR is the so-called “water hammer effect” caused by the increased pressure of urine on the tubular orifices located at the papillary surface [34,35]. According to Funston et al., it seems that IRR is more likely to appear under less urinary pressure in very young children than in older ones, but the pressure for the occurrence of IRR is growing with age. The increased urine pressure and the occurrence of IRR can be expected in patients with functional or obstructive urinary tract anomalies, especially in anomalies of the urinary bladder that can increase intravesical urinary pressure [52]. As the IRR appears mostly in very young children with a strong tendency for disappearance after the fourth year of life, we could speculate that, during kidney growth, the orifices of papillary ducts get narrower and shrink probably as a consequence of the specific renal response to infection or other inflammatory processes associated with the backflowed urine into the tubulointerstitial space [10,34,53,54,55,56]. These factors possibly cause the induction of cellular proliferation, which leads to the cessation of the urinary backflow through the papillary tubuli.

### 2.1. The Diagnostic Methods for the Detection of IRR: VCUG

After the first description of IRR, it has been noticed and described many times by using VCUG with an incidence of 1–11% out of all diagnosed VURs. However, the incidence depended on the technique of the VCUG procedure. In the study by Schneider KO et al., the authors claimed that the prevalence of IRR diagnosed by VCUG should be higher than 20% if using the improved VUR technique. They found the prevalence of IRR in patients with a grade IV VUR at 27.4% and 44.4% in a VUR of grade V. Taking into account all grades of VUR together, the prevalence of IRR was 11%. Usually, the prevalence of IRR in most other studies was about 2% [46,47,49,50,51].

**Figure 2:**
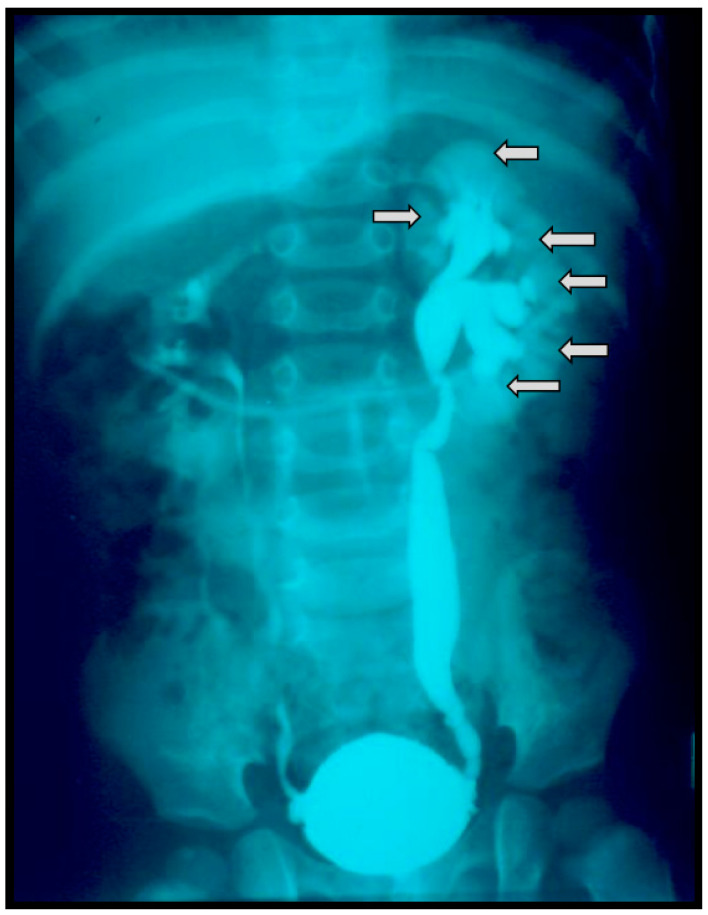
Voiding cystourethrography shows the bilateral vesicouretral reflux of grade II on the right side and of grade III on the left side. In the parenchyma of all three segments of the left kidney, the contrast media is visible, presenting intrarenal reflux (arrows).

Some authors suggested that IRR occurs exclusively in the highest grades of VURs, like in the study by Schneider et al., which reported that 100% of described IRRs belonged to the group of dilated VURs. In contrast, other groups of authors reported that up to 14% of all described IRRs belonged to the group of non-dilated VURs when VCUG was used as a diagnostic tool [35,53,54,57,58,59,60,61]. The presence of IRR, diagnosed by VCUG, was very often correlated with the extent of parenchymal damage, especially in patients with high grades of VUR-s (Figure 2). Parenchymal changes were mostly described as renal scars, photon defects, or decreased differential renal function (<40%) diagnosed by DMSA scan, or as the signs of renal scarring if the IVU was used [28,53,61]. Parenchymal changes corresponded very well to the sites of IRR and were presented mostly by parenchymal scars. In the studies where the VCUG and DMSA scans were used, up to 65% of kidneys affected by IRR tended to develop renal scars gradually during this time [34,35,54,61]. That means that the formation of scars is a continuous and possibly unrestrainable independent process. Some authors found a strong correlation between the position of IRR and parenchymal defects [33,58,60]. The relationship between IRR, urinary tract infection, and parenchymal scarring has been established in some previous studies as well [6,53,61].

In summary, according to numerous studies, we could conclude that predominantly high-grade VURs with IRR were associated with parenchymal damage rather than those with lower grades of VUR if the VCUG was used. However, there was also the group of kidneys with IRR that were not affected by parenchymal changes at the time of examination [35,54,61].

### 2.2. The Diagnostic Methods for the Detection of IRR: ceVUS

Nowadays, the diagnosis of VUR, as well as the diagnosis of IRR, is possible without radiation burden by using the ceVUS method, especially with the second generation of contrast media [9,10,55,62,63,64,65,66]. The use of ceVUS opened new possibilities for the diagnosis of IRR, showing that the incidence of IRR appeared in a range between 11.9 and 61.5% out of all diagnosed VURs [9,10,55,64,65,66]. The study by Simičić A. et al. showed that 100% of VURs grade IV and V and 83.8% of VURs grade III had IRR. Moreover, 14.5% of VURs grade II had IRR, while the overall percentage of IRR was 49.5%. Since the VURs of grade II were highly represented in that study, they accounted for 21% of all diagnosed IRRs [10]. The existence of IRRs in the group of non-dilated VURs was also noticed in some other similar studies [9,10,55,64], while another group of studies reported the opposite results [62,63,65,66]. We believe that such differences regarding the percentage of IRRs in the group of non-dilating VURs might be explained by a small number of cases in some studies, deficiencies in diagnostic procedures, or differences in the used equipment. During the existence of IRR, the kidneys are propensed to UTI, which probably leads to certain consequences, including the full spectrum of parenchymal changes that can be detected by DMSA scan [9,53,61,63,67].

## 3. A Comparison of IRRs Diagnosed by VCUG and ceVUS

As the ceVUS diagnoses a much higher number of cases of IRRs than VCUG [9,10,55,63,66], that information needs additional explanation regarding the influence of the IRR on renal parenchyma. One of the previous studies performed by VCUG showed that 76% of IRRs were associated with photon defects, with a strong tendency for scar formation in 65% of cases, particularly at the sites of IRR when analyzed by the DMSA scan [61]. Another study, using the same methodology, also showed that the share of kidneys with a differential renal function lower than 40% was significantly higher represented in the group of kidneys with IRR than in the control group [59]. That means that IRRs discovered by VCUG were associated with a higher percentage of parenchymal changes than those discovered by ceVUS [9]. The possible explanation for that could be that IRR-associated VURs were discovered in a relatively small portion by VCUG, mostly in the group of highest grades of VURs, where the parenchymal damage was expected. By using the VCUG method, it was only possible to conclude that IRRs accompanied by high-grade VURs had a gradual development of scars [53,54,59].

According to Fujimatsu et al., low-grade VURs accompanied by IRR were not connected with parenchymal changes, while in contrast, Kim SW et al. found photon defects on DMSA scans in 25% of that group of patients [54,61]. In the new circumstances of the high sensitivity of ceVUS, which detects 5–50 times more IRRs than VCUG, it became very interesting to establish how many newly detected IRRs by ceVUS would develop parenchymal defects on DMSA scan and what type of parenchymal changes would be detected [9]. Additionally, IRRs detected by ceVUS were expected to be associated with changes in DMSA scan, but with different results than in studies where the IRRs were found by VCUG [9,53,59,61]. The prevalence of parenchymal defects on the DMSA scan was expected to be higher at the sites of IRRs detected by VCUG than in those detected by ceVUS because the number of IRRs detected by ceVUS appears many times more frequent than those detected by VCUG. On the other hand, the DMSA study as the independent method always shows the same number and extent of changes in the renal parenchyma, regardless of the method used for the detection of VUR and IRR. In the patients examined by ceVUS, those parenchymal changes are divided among a high number of newly diagnosed IRRs within the affected kidneys. At the same time, we believe that many of the locations in renal parenchyma, spotted on a DMSA scan in kidneys with VURs without IRR correlated with undiagnosed IRRs detected by VCUG. As the VCUG detects a small number of IRRs associated with high grades of VUR, the corresponding DMSA scan findings mostly reflect the more advanced parenchymal defects like scars or rarely gradual changes like diminished uptake of the radiotracer [33,54,59,61]. That correlation of parenchymal defects with the site of IRR diagnosed by VCUG and DMSA scan disclosed mostly the final consequences in the renal parenchyma that are unsuitable for any treatment, and that was disappointing for a therapeutic approach to IRR-associated VURs.

The portion of IRR places that were still unaffected by parenchymal changes on the DMSA scan was 24% in the study by Kim SW et al. performed by VCUG [61]. From that point of view, at the sites of IRRs diagnosed by ceVUS, a wide spectrum of parenchymal defects was noticed in the study by Simičić et al. Namely, 38% of diagnosed IRRs were affected with a wide spectrum of parenchymal changes, but with a relatively small number of definite scars (13.6%), and a rather high percentage (62%) of IRRs that were not associated with parenchymal changes at all. The results of that study opened a possible new perspective for the diagnostics and treatment of patients affected by IRR-associated VURs [9]. That was the only study performed by a late DMSA scan combined with ceVUS. The survey by Oh S from 2022 performed by ceVUS showed photon defects in 79% of IRRs by acute DMSA scan, which was done during the acute infection. It, therefore, was not comparable to other studies that used the DMSA scan at least three months after febrile UTI [66]. Thus 62% of the cases diagnosed with ceVUS were still preventable regarding scar development by antibiotic therapy, long-term antibiotic prophylaxis (LTAP), or surgical treatment [9]. Moreover, all the kidneys with IRRs had significantly more parenchymal changes compared to those without IRRs, regardless of the grade of VUR. All IRR-associated VURs have a potential risk for the development of renal injury, regardless of the VUR grade, and they have to be treated equally [9].

That fact could substantially change the existing paradigm of therapeutic approach, especially regarding the application of LTAP, hoping that unaffected parts of renal parenchyma in kidneys with IRR would remain without parenchymal changes. That also implies that we have a firm argument in favor of the introduction of LTPA in patients with IRR diagnosed by ceVUS. Moreover, in addition to the antibiotic treatment, we expect that immunosuppressive therapy could be applied in the future for the prevention of the possible final pathohistological consequences of IRR [54]. According to those facts, we think that we have now a new reason to treat all IRR-associated VURs, including grade II by LTAP or by surgery in the same way as IRR-associated VURs grade IV or V, which has not been previously recommended [47,68,69,70,71]. The previous studies did not take into consideration the existence of IRR as an important factor that could influence the treatment modalities and recommendations for patients with VUR and UTI [47,68,69,70,71]. Nowadays, we know that in addition to high-grade VURs, diagnosed by ceVUS, which are highly accompanied by IRRs, up to 20% of all IRRs belong to the VUR grade II and that they could also be prone to the development of parenchymal changes [9]. The recent data revealed that problem in a new way, indicating the new treatment opportunities for the prevention of renal parenchymal damage caused by UTI, VUR, and IRR together. Because 62% of IRRs are still unaffected by parenchymal changes, we believe that there is still plenty of room for preventional LTAP or even surgical treatment until the VUR or IRR disappears.

Before the development of ceVUS, the authors of previous studies gave controversial recommendations regarding the treatment options of all grades of VURs [9,10]. Those recommendations included the treatment of exclusively the high-grade VURs accompanied by febrile UTI, extremely young patients with VUR, patients affected with Pseudomonas aeruginosa, and patients with bladder–bowel dysfunction by antibiotics and surgery, while some other groups of patients were recommended to exclude LTAP [47,68,69,70,71]. Some authors recommended not treating VURs with LTAP or even not diagnosing VURs at all [68].

According to the actual data, we could propose that all patients with IRRs, including those with VUR grade II, should be included in the program of LTAP. That discussion about who would be recruited into LTAP or not was carried on for a long time. We believe that the new level of information will give a new guidepost for making conclusions and decisions regarding therapeutic options. Thus, while IRR-associated VURs belong to the risk group for the development of parenchymal defects and scars, VURs that are not associated with IRR do not belong to that risk group, so they should be treated only during the acute UTI by antibiotics and not protected by LTAP. Additionally, we believe that it is extremely important to treat and protect patients with IRR-associated VURs during the first five years of life, especially during the first year of life. Namely, we expect that IRRs will be healed in most patients up to five years of life, when the LTAP will not be necessary anymore [12,13,34,60,64]. Another risk factor for renal damage is surely the sex because boys are affected with IRRs more frequently than girls, with significantly earlier clinical presentation than in girls and a higher percentage of dilated VURs associated with IRR [12].

That conclusion would be effective only if the patients are examined by ceVUS, which is the superior diagnostic method for detecting VUR and IRR rather than VCUG, thus allowing us to locate the sites of potential parenchymal changes that highly correlate with the locations of compound papillae types II and III [12,13,51]. According to the former state of knowledge, based on VCUG, 65% of IRRs were presented with scars that were out of reach of antibiotic therapy. From that point of view, the therapy was questionable and had a limited effect, except in a small proportion of IRRs without the full development of the final consequences [34,61]. In contrast, the study by Simičić Majce et al. showed many times more IRRs by ceVUS than by VCUG but only 38% of parenchymal defects at the site of IRR. These defects were mainly mild, presented by diminished and inhomogeneous uptake of DMSA, and rarely developed scars [12]. That means that 62% of IRRs are left unaffected at the time of diagnosis and should be highly recommended to LTAP.

Although the previous studies based on VCUG findings did not find a strong motive to include IRRs in the classification of VURs, with improved knowledge and the implementation of ceVUS, we believe that the presence of IRR should be included in the future classification of VUR. The main goal is to separate the cases of VURs with high risk for the development of parenchymal changes from those who are not at risk [9]. By that classification, it will be possible to reduce the number of patients on LTAP and, at the same time, to include targeted groups of patients in LTAP to prevent definite parenchymal changes.

## 4. The New Propositions for the Inclusion of Patients with IRR in LTAP

According to the actual data, we could propose that all patients with IRRs, including those with VUR grade II, should be included in the program of LTAP. That discussion about who would be recruited into LTAP or not was carried on for a long time. We believe that the new level of information will give a new guidepost for making conclusions and decisions regarding therapeutic options. Thus, while IRR-associated VURs belong to the risk group for the development of parenchymal defects and scars, VURs that are not associated with IRR do not belong to that risk group, so they should be treated only during the acute UTI by antibiotics and not protected by LTAP. Additionally, we believe that it is extremely important to treat and protect patients with IRR-associated VURs during the first 5 years of life, especially during the first year of life. Namely, we expect that IRRs will be healed in most patients up to 5 years of life, when the LTAP will not be necessary anymore [9,10,34,60,64]. Another risk factor for renal damage is surely sex because boys are affected with IRR more frequently than girls, with significantly earlier clinical presentation than in girls, and have a higher percentage of dilated VURs associated with IRR [9]. That conclusion would be effective only if the patients are examined by ceVUS, which is the superior diagnostic method for detecting VUR and IRR than VCUG, thus allowing us to locate the sites of potential parenchymal changes that highly correlate with the locations of compound papillae type II and III [9,10,51].

According to the former state of knowledge, based on VCUG, 65% of IRRs were presented by scars that were out of reach of antibiotic therapy. From that point of view, the therapy was questionable and had a limited effect, except in a small proportion of IRRs without the full development of the final consequences [34,61]. In contrast, the study by Simičić Majce et al. showed many times more IRRs by ceVUS than by VCUG, but only 38% of parenchymal defects at the site of IRR. These defects were mainly mild, presented by diminished and inhomogeneous uptake of DMSA, and rarely developed scars [9]. That means that 62% of IRRs are left unaffected at the time of diagnosis and should be highly recommended to LTAP. Although the previous studies, based on VCUG findings, did not find a strong motive to include the IRR in the classification of VURs, with the improved knowledge contributed by the implementation of ceVUS, we believe that the presence of IRR should be included in the future classification of VUR. The main goal is to separate the cases of VURs with a high risk for the development of parenchymal changes from those who are not at risk [6]. By that classification, it will be possible to reduce the number of patients on LTAP and, at the same time, to include targeted groups of patients in LTAP to prevent definite parenchymal changes. A new long-term double-blind prospective study on patients with IRR with the inclusion of a placebo group will be needed to confirm or decline the idea of the new therapeutical approach to patients with IRR-associated VURs.

## 5. Conclusions

In that sense, we propose the new classification of VURs according to the ceVUS methodology (Table 1).

Based on new information that was based on the application ceVUS method, we propose LTAP for the patients with VUR grades II A, III A, IV A, and V A until the spontaneous resolution of IRR or successful surgical intervention. The choice of LTAP agents should be based on the local reports of resistance rates of uropathogenic bacteria to antibiotics, if possible, because it is well known that, in some regions, some antibiotics are not in use anymore due to the high resistance rate [72].

At the very end of these conclusions, we could say that due to the high sensitivity of ceVUS in the detection of IRRs that show a high possibility for the development of parenchymal changes, we should reconsider the use of DMSA scan application for the future detection of the parenchymal changes because of the radiation that DMSA gives to the patient.

## Figures and Tables

**Table 1:** A new classification of VUR according to ceVUS.

Grade	Description
I	The contrast is visible only in the distal part of the ureter.
II	The contrast is visible in the ureter and renal cavities, without dilatation.
II A	The contrast is visible in the ureter and renal cavities, without dilatation and with intrarenal reflux in the upper, lower, or middle renal segments, or in combination with the segments.
III	The contrast is visible in the ureter and renal cavities with mild dilatation
III A	The contrast is visible in the ureter and renal cavities, with mild dilatation and with intrarenal reflux in the upper, lower, or middle renal segments, or in combination with the segments.
IV	The contrast is visible in the ureter and renal cavities with moderate dilatation.
IV A	The contrast is visible in the ureter and renal cavities, with moderate dilatation and with intrarenal reflux in the upper, lower, or middle renal segments, or in combination with the segments
V	The contrast is visible in the highly dilated and tortuous ureter and the highly dilated and deformed renal cavities with convex calyces.
V A	The contrast is visible in the highly dilated and tortuous ureter and the highly dilated and deformed renal cavities, with convex calyces and with intrarenal reflux in the upper, lower, or middle renal segments with segments or in combination

## Data Availability

Data will be available upon request.
